# Dual-factor model of sleep and diet: a new approach to understanding central fatigue

**DOI:** 10.3389/fnins.2024.1465568

**Published:** 2024-09-17

**Authors:** Yifei Zhang, Zehan Zhang, Qingqian Yu, Bijuan Lan, Qinghuan Shi, Yan Liu, Weiyue Zhang, Feng Li

**Affiliations:** ^1^School of Tradional Chinese Medicine, Beijing University of Chinese Medicine, Beijing, China; ^2^School of Nursing, Beijing University of Chinese Medicine, Beijing, China

**Keywords:** central fatigue, high-fat diet, alternate-day fasting, animal model, rats, oxidative stress, cognitive function alterations

## Abstract

**Background:**

Numerous studies have recently examined the impact of dietary factors such as high-fat diets on fatigue. Our study aims to investigate whether high-fat diet (HFD) alone or combined with alternate-day fasting (ADF) can lead to the central fatigue symptoms and to investigate the potential integration of dietary and sleep variables in the development of central fatigue models.

**Methods:**

Seventy-five male Wistar rats were divided into five groups: control, HFD, HFD + ADF, modified multiple platform method (MMPM), and MMPM+HFD + ADF. Each group underwent a 21-day modeling period according to their respective protocol. Their behavioral characteristics, fatigue biochemical markers, hippocampal pathological changes, mitochondrial ultrastructure, and oxidative stress damage were analyzed.

**Results:**

Our findings demonstrate that using only HFD did not cause central fatigue, but combining it with ADF did. This combination led to reduced exercise endurance, decreased locomotor activity, impaired learning and memory abilities, along with alterations in serum levels of alanine aminotransferase (ALT), creatine kinase (CK), and lactate (LAC), as well as hippocampal pathological damage and other central fatigue symptoms. Moreover, the MMPM+HFD + ADF method led to the most obvious central fatigue symptoms in rats, including a variety of behavioral changes, alterations in fatigue-related biochemical metabolic markers, prominent pathological changes in hippocampal tissue, severe damage to the ultrastructure of mitochondria in hippocampal regions, changes in neurotransmitters, and evident oxidative stress damage. Additionally, it was observed that rats subjected to HFD + ADF, MMPM, and MMPM+HFD + ADF modeling method exhibited significant brain oxidative stress damage.

**Conclusion:**

We have demonstrated the promotive role of dietary factors in the development of central fatigue and have successfully established a more stable and clinically relevant animal model of central fatigue by integrating dietary and sleep factors.

## 1 Introduction

Fatigue may be distinguished as either central fatigue or peripheral fatigue, dependent upon the primary site of its manifestation (Yang et al., 2022). The manifestations of central fatigue are nuanced, encompassing a spectrum of aspects including psychological dispiritedness, diminished appetite, lethargy, and reduced cognitive acuity (Kwak et al., 2020; Lee et al., 2022). Presently, the etiological intricacies of central fatigue remain enigmatic (Rasmussen et al., 2007).

Animal models are currently utilized to investigate the mechanisms of fatigue, with a particular focus on central fatigue (Lu et al., 2022). Studies conducted to date have clarified that the Modified Multiple Platform Method (MMPM), a prominent method of sleep deprivation, is frequently employed and has proven effective in the development of central fatigue models (Han C, et al., 2017). However, this animal model exclusively considers the impact of individual sleep factors on the emergence of central fatigue, neglecting to incorporate other factors that may contribute to central fatigue.

An optimal animal model for fatigue should ideally encompass a broad spectrum of attributes that reflect the human experience of fatigue (Moriya et al., 2015). In addition to sleep factors, clinical studies have established a significant correlation between poor dietary habits and both self-reported and subjective fatigue symptoms (Park et al., 2013; Ikeda et al., 2011). Current research suggests that a high-fat diet (HFD) may limit carbohydrate intake, thereby impeding glycogen synthesis and precipitating the emergence of fatigue symptoms (Nosaka et al., 2020). In animal models, the intake of a high-fat diet can result in manifestations potentially associated with both physical and mental fatigue, as well as changes in metabolic substances (Liu et al., 2023; Han X, et al., 2017; Underwood and Thompson, 2016; Wang et al., 2018).

In addition, it is widely acknowledged that both acute and chronic fasting can trigger episodes of binge eating and overeating, disrupting normal eating patterns and leading to the occurrence of irregular eating habits (Mbalilaki et al., 2010; Ge et al., 2020). However, to date, no studies have considered the factor of irregular diet in central fatigue animal models, and there is also a lack of research exploring the potential combined effects of irregular diet and sleep factors.

In this study, our aim is to explore whether HFD alone or in combination with ADF can elicit central fatigue symptoms in rats. Furthermore, building upon the existing Modified Multiple Platform Method (MMPM) animal model, our research seeks to develop a dual-factor model that integrates sleep and dietary factors (by integrating HFD, ADF, and MMPM) to more accurately reflect clinical pathogenesis, and to investigate whether this model results in disruptions to energy metabolism and oxidative stress-induced brain damage in model rats.

## 2 Materials and methods

### 2.1 Animals

Seventy-five male specific pathogen-free (SPF) Wistar rats weighing 200 ± 20 g were procured from the Beijing SPF Biotechnology Co., Ltd. (license number: SCXK 2019–0010). Experimental animals were maintained in the Beijing University of Traditional Chinese Medicine animal experimental center (5 rats per cage, 12 h of light/dark alternation, temperature 23 ± 2°C, humidity 35% ± 5%, and free drinking water). The animal protocol was approved by the Animal Experimentation Ethics Committee of Beijing University of Chinese Medicine (number: BUCM-2024051601-2114). Before the experiments, all Wistar rats were placed in the SPF experimental environment for 7 days for acclimation. During the adaptive feeding period, all rats had free access to drinking water and were provided with a standard maintenance diet.

### 2.2 Animal grouping and model establishment

Utilizing computer-generated random number sequences, 75 rats were allocated into 5 groups: control group, HFD group, HFD + ADF group, MMPM group, and MMPM+HFD + ADF group, with 15 rats per group. It is necessary to ensure that there are no significant differences in general conditions such as body weight, water intake, mental state and activity among the five groups of rats.

**Figure 1:**
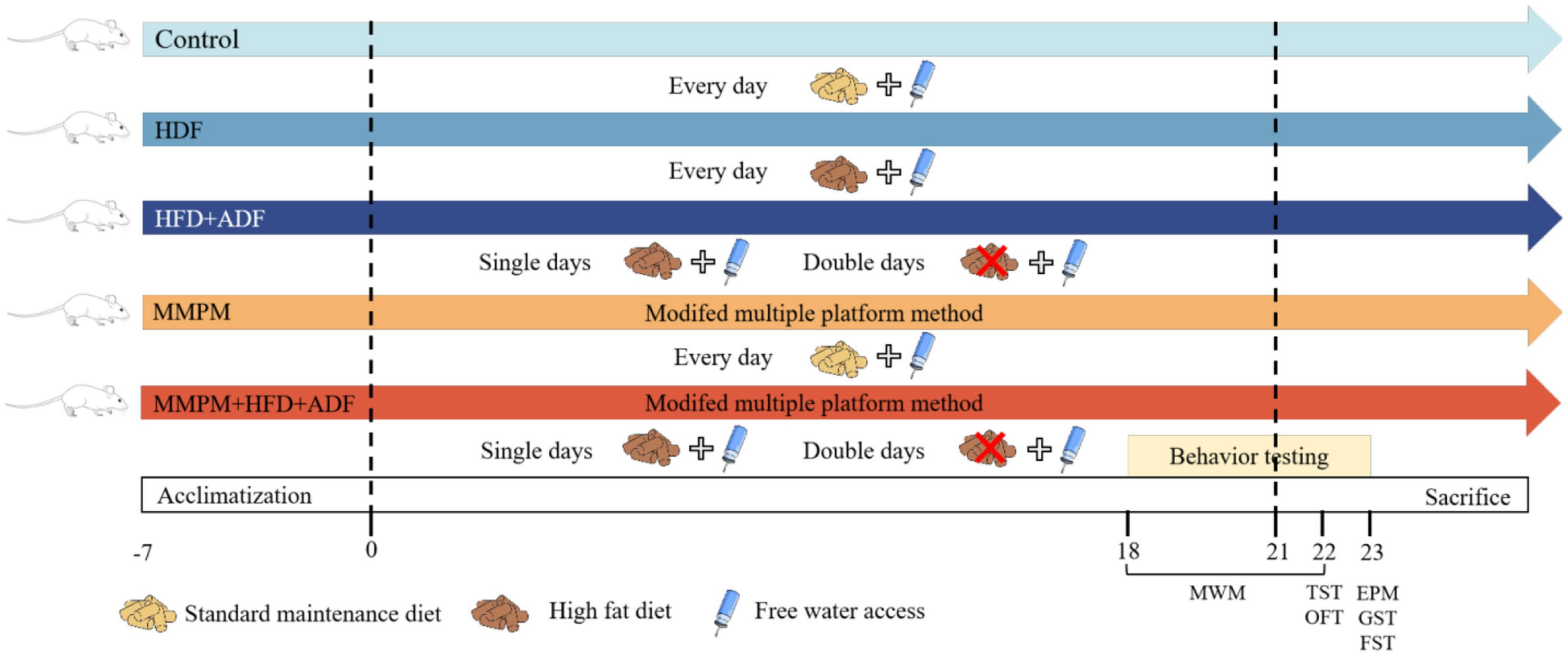
Experimental procedure. MWM, Morris Water Maze; TST, Tail Suspension Test; OFT, Open Field Test; EMP, Elevated Plus Maze; GST, Grip Strength Test; FEST, Forced Exhaustive Swimming Test; Single days, each odd-numbered day in the experimental process, such as Day 1, Day 3, up to Day 21; Double days, each even-numbered day in the experimental process, such as Day 2, Day 4, up to Day 20; MMPM, Modified Multiple Platform Method; HFD, high-fat diet; ADF, alternate-day fasting.

After the beginning of the experiment, standard maintenance diet was subjected to rats in the blank control group. Rats in the HFD group were fed with HFD for 21 days (The HFD used in the experiment was provided by Beijing SPF Bio-technology Co., Ltd., Cat # SFD005, consisting of 83.5% base feed, 15% lard, and 1.5% cholesterol.). The modeling method of rats in the HFD + ADF group was single-day fasting and double day HFD for 21 days. MMPM group mainly refers to the previous modeling method of our team (Zhang et al., 2018). Rats were placed on a stationary water environment platform from 6 p.m. to 8 a.m. the following day, undergoing modeling for 14 h daily for a total of 21 days. Rats in the MMPM+HFD + ADF group were placed on the platform each evening at 6 p.m., remaining for 14 h until removal the following morning at 8 a.m. On odd-numbered days, the rats were provided with a high-fat diet and water; on even-numbered days, they were fasted, receiving only water. This model method was lasting for 21 days. The experimental schematic is shown in Figure 1.

### 2.3 Behavioral testing

#### 2.3.1 Grip strength test

The grip strength experiment is a commonly used method to assess the neuromuscular function in rodents (Aldhahri et al., 2022). On the 23rd day of the whole experiment, each rat was gently placed onto the lever of the grip strength meter (developed by Zhongshi Di Technology Development Co., Ltd., Beijing, China), where they grasped the lever with their forepaws, and their grip strength was quantified by electronic sensors attached to the lever. Gently grasping the tail of the rat with one hand, a uniform force was steadily applied in a backward direction until the rat released its grip from the outermost layer of the lever. The maximum grip strength value of each rat was recorded. Each rat underwent three consecutive trials, and the average value of the three trial results was calculated for subsequent statistical analysis (Zhu et al., 2022).

#### 2.3.2 Forced exhaustive swimming test

The FEST is frequently utilized to assess the fatigue levels in rodent models (Chen et al., 2019). On the 23rd day of the whole experiment, we attached a lead wire ring, weighing approximately 10% of the rat’s body weight, to the rat’s tail using a thin rope (Tang et al., 2008). The placement was positioned between one-third to two-thirds distance from the base of the tail. With a gentle grasp on the rat’s dorsal region, we placed the rat with its head oriented upwards into a swimming tank, forcing the rats to swim with the added weight. Commencing the timing as the rat enters the water, exhaustion time were recorded when the rat fails to resurface within 10 s after submerging.

#### 2.3.3 Open field test

The OFT is a commonly used rodent behavioral experiment, which was utilized to evaluate locomotor activity of rats (Knight et al., 2021). The OFT was performed on the 22nd day of the experiment. Briefly, a high-definition camera was placed in a dimly-lit room above an open field box (100 cm × 100 cm × 40 cm, provided by Beijing Zhongshi Di Development Co., Ltd.) to record the movement trajectory. The rats were swiftly placed into the center of the open field box from the same direction, initiating both image capture and timing simultaneously. The autonomous activities of the rats were continuously observed for 5 min, during which the duration of rearing and grooming within 5 min was recorded. Furthermore, total distance and central percentage were analyzed using an animal motion tracking system (Noldus, Netherlands).

#### 2.3.4 Elevated plus maze test

The elevated plus maze test was employed to assess anxiety-like behavior in rats (Bruijnzeel et al., 2019). On the 23rd day of the whole experiment, the elevated plus maze (100 cm × 100 cm × 40 cm, provided by Zhongshi Di Technology Development Co., Ltd., Beijing, China) was placed under a high-definition camera. The rats were positioned facing one direction on the central platform, with their heads oriented toward an open arm of the maze, initiating both image acquisition and timing simultaneously. The spontaneous activities of the rats within a 5-min interval were continuously monitored, and the animal motion tracking system was employed to analyze the percentage of entries into the open arms as well as the time spent within the open arms (Hilton et al., 2023).

#### 2.3.5 Tail suspension test

The tail suspension test was used to evaluate depression-like behavior and fatigue in rats (Zhang et al., 2023; Zhang et al., 2022). Tail suspension tests were performed as previously described (Galeotti and Ghelardini, 2012). On the 22nd day of the whole experiment, the rats’ tails were threaded through a circular hole at the center of an opaque partition with an approximate diameter of 1.5 cm. Subsequently, the upper third of the tail base was secured to the partition, causing the rats to be suspended upside down with the tail upward and the head downward for a continuous observation period of 6 min. The immobility duration and struggle frequency were monitored within 4 min after the observation period (Zhang et al., 2023).

#### 2.3.6 Morris water maze test

The Morris water maze test is a classic assay used to evaluate spatial learning and memory in rodents (Barnhart et al., 2015). The water pool (provided by Zhongshi Di Development Co., Ltd., Beijing, China) used in our test was 1 m in diameter and made of black polyvinyl chloride. The water pool was filled with water maintained at a temperature of 25 ± 1°C. The pool was evenly divided into four quadrants along the circumference, with a movable platform placed in the center of the first quadrant and secured in place. The whole test lasted for 5 days, including a 4-day learning phase and a 1-day formal test phase. On each day of the learning phase, make sure that all the rats have access to the pool from each of the four entry points, and the sequence of entry points changes daily. After the training phase, on the 22nd day of the experiment, the formal test was carried out by removing the movable platform from the pool. Each rat was placed into the pool from the entry point in the third quadrant, and the movement of the rat in the pool was recorded for 120 s. The animal motion tracking system was used to analyze escape latency, platform crossings, time spent in the target quadrant during the formal test phase.

### 2.4 Serum biochemical index detection

Following a 12-h period of fasting, the rats were humanely euthanized utilizing 3% sodium pentobarbital (3 mL/kg, Cat #0894–5 g, Sigma) as an anesthetic. Subsequently, approximately 5 mL of blood was extracted from the abdominal aorta into blood collection tubes without anticoagulants. The obtained blood was left undisturbed in the collection tubes for a duration of 2 h, following which the serum was isolated through centrifugation at 3000 × g for 10 min at a temperature of 4°C. The concentrations of aspartate aminotransferase (AST), alanine aminotransferase (ALT), blood urea nitrogen (BUN), lactate (LAC), lactate dehydrogenase (LDH), creatine kinase (CK), and glucose (GLU) in the serum were quantified employing an automated biochemical analyzer (Beckman Coulter, Brea, CA, United States).

### 2.5 Hematoxylin/eosin staining

The rats were anesthetized with 3% pentobarbital sodium and then perfused through the heart with 4% paraformaldehyde. The entire brain of the rats was extracted, fixed in 4% paraformaldehyde (Cat#1101, China) for over 24 h, and subsequently processed for preparation into paraffin sections. The sections were processed according to the following protocol: deparaffinized with xylene, hydrated with gradient alcohol, stained with hematoxylin, differentiated with hydrochloric alcohol, stained with eosin and dehydrated in a graded alcohol series, xylene transparency, and neutral gum sealing. The pathological changes in the hippocampal tissues of each group of rats were observed under a conventional light microscope (Nikon, Japan).

### 2.6 Nissl staining

Nissl staining was used to identify nissl bodies in the neuronal cytoplasm and detect neuronal injury (Shi et al., 2021; Liu et al., 2022). The entire brain was extracted from rats perfused with 4% paraformaldehyde through the heart, followed by preparation of paraffin sections and subsequent Nissl staining. Following dewaxing, gradual alcohol hydration, and staining with 0.5% cresyl violet (Cat #B1007, Baiqiandu Biological Technology, Ltd., China), the sections were dehydrated, clarified with xylene, and mounted with neutral resin. Subsequently, the pathological damage of Nissl bodies in the hippocampus of the rats was observed under a conventional light microscope (Nikon, Japan).

### 2.7 Enzyme-linked immunosorbent assay

The levels of glycogen, adenosine triphosphate (ATP), adenosine diphosphate (ADP), 5-hydroxytryptamine (5-HT), and dopamine (DA) were measured using the enzyme-linked immunosorbent assay (ELISA) method. Following the manufacturer’s instructions, muscle glycogen levels and hepatic glycogen levels were determined using Muscle Glycogen Assay Kit (Cat #MB-7562A, Enzyme Biotech, China) and Liver Glycogen Assay Kit (Cat #MB-22066A, Enzyme Biotech, China), respectively. Furthermore, the ATP and ADP levels in the hippocampus were quantified using the ATP assay kit (Cat #MB-6913A, Enzyme Biotech, China) and ADP assay kit (Cat #MB-6956A, Enzyme Biotech, China). Moreover, the levels of 5-HT and DA in the brain hippocampal tissue were quantitated utilizing the 5-HT assay kit (Cat #MB-1983A, Enzyme Biotech, China) and the DA assay kit (Cat #MB-1896A, Enzyme Biotech, China) following the manufacturer’s instructions.

### 2.8 Determination of SOD, MDA and GSH-Px levels in the hippocampus

The levels of superoxide dismutase (SOD), malondialdehyde (MDA), and glutathione peroxidase (GSH-Px) in the hippocampal tissue of rats are utilized to assess the extent of oxidative stress damage in the brains of fatigued rats. The determination of SOD activity was carried out using a Total Superoxide Dismutase Assay kit (Cat #S0101S, Beyotime, Shanghai, China). Following tissue sample lysis, the supernatant was collected after centrifugation at 12,000 × g for 5 min at 4°C, then mixed with a reaction starter reagent and incubated at 37°C for 30 min. The SOD activity was determined by reading the absorbance at 450 nm. The quantification of MDA levels was measured by a Lipid Peroxidation MDA Assay kit (Cat #S0131S, Beyotime, Shanghai, China). Following tissue lysis, the supernatant was obtained after centrifugation at 12,000 × g for 10 min at 4°C. The supernatant was then mixed with a working fluid, heated in a water bath at 100°C for 15 min, cooled to room temperature, centrifuged at 1,000 × g for 10 min, and absorbance was measured at 532 nm. The activity of GSH-Px was assessed using a Cellular Glutathione Peroxidase Assay Kit with DTNB (Cat #S0057S, Beyotime, Shanghai, China). After tissue lysis and centrifugation for 10 min, the supernatant was mixed with a working fluid and incubated at room temperature for 10 min, followed by a further incubation with DTNB solution at room temperature for an additional 10 min. Absorbance was measured at 412 nm to calculate enzyme activity.

### 2.9 Observation of mitochondrial ultrastructure

The ultrastructure of the mitochondria in hippocampal CA1 region was observed under a transmission electron microscope. After sacrificing the rats, fresh tissue samples of the hippocampal CA1 region (1 mm × 1 mm × 1 mm in volume) were swiftly fixed in electron microscope fixation liquid (Cat #B0012, Baiqiandu Biological Technology, Ltd., China) at 4°C for 2–4 h. Subsequently, the samples were fixed at 20°C in 0.1 M phosphate-buffered saline (PBS, pH 7.4) for 2 h, followed by three washes, graded alcohol dehydration, infiltration, and embedding. The samples were then sectioned into 60-80 nm ultrathin slices using an ultramicrotome (Leica, Shanghai, China). The sections were stained and air-dried overnight at room temperature. The Flameng score was employed as a quantitative measure to evaluate the degree of mitochondrial damage. A total of three randomly selected fields were examined in each sample, with 10 mitochondria observed within each field.

### 2.10 Reactive oxygen species levels detection

In order to quantify the levels of ROS in the rat hippocampal tissue, frozen sections of fresh rat brain hippocampus tissue were thawed. Subsequently, a circle was delineated around the tissue using a tissue-marking pen for histological assessment. A freshly prepared dihydroethidium (DHE) staining solution (Cat #D7008, Sigma, China), diluted in phosphate-buffered saline (PBS) solution (Cat #G0002, Baiqiandu Biological Technology, Ltd., China), was administered onto the tissue. The samples were subsequently incubated at 37°C protected from light for 30 min (Rao et al., 2020), followed by destaining. Then, added 4′,6-diamidino-2-phenylindole (DAPI) staining solution (catalog number B0011, Baiqiandu Biological Technology, Ltd., China) and incubated at room temperature for 10 min in the dark. Following destaining, the slides were dried and sealed with anti-fluorescence quenching sealing tablets (Cat #B1401, Baiqiandu Biological Technology, Ltd., China). A fluorescence microscope was utilized to capture the image. The software Image-Pro Plus 6.0 was employed for the determination of integrated optical density (IOD) values, from which the average luminance density value was calculated by IOD/AREA.

### 2.11 Statistical methods

All statistical analyses were performed utilizing the SPSS 26.0 software (IBM, Armonk, NY, United States), and GraphPad software (version 9.5.0, San Diego, CA, United States) was utilized for the creation of relevant graphs based on the statistical results. For data with a normal distribution, one-way ANOVA was used for group comparisons, LSD test or Tamhane test were used for pairwise comparison while Kruskal-Wallis test was used for non-normal data distribution. A *p*-value <0.05 denoted a statistically significant different between the values. All behavioral tests, histopathology, electron microscopy, and assays were conducted by blinded observers. All data is expressed as the mean ± standard deviation (mean ± SD).

## 3 Results

### 3.1 The spontaneous activity and physical fatigue status

**Figure 2:**
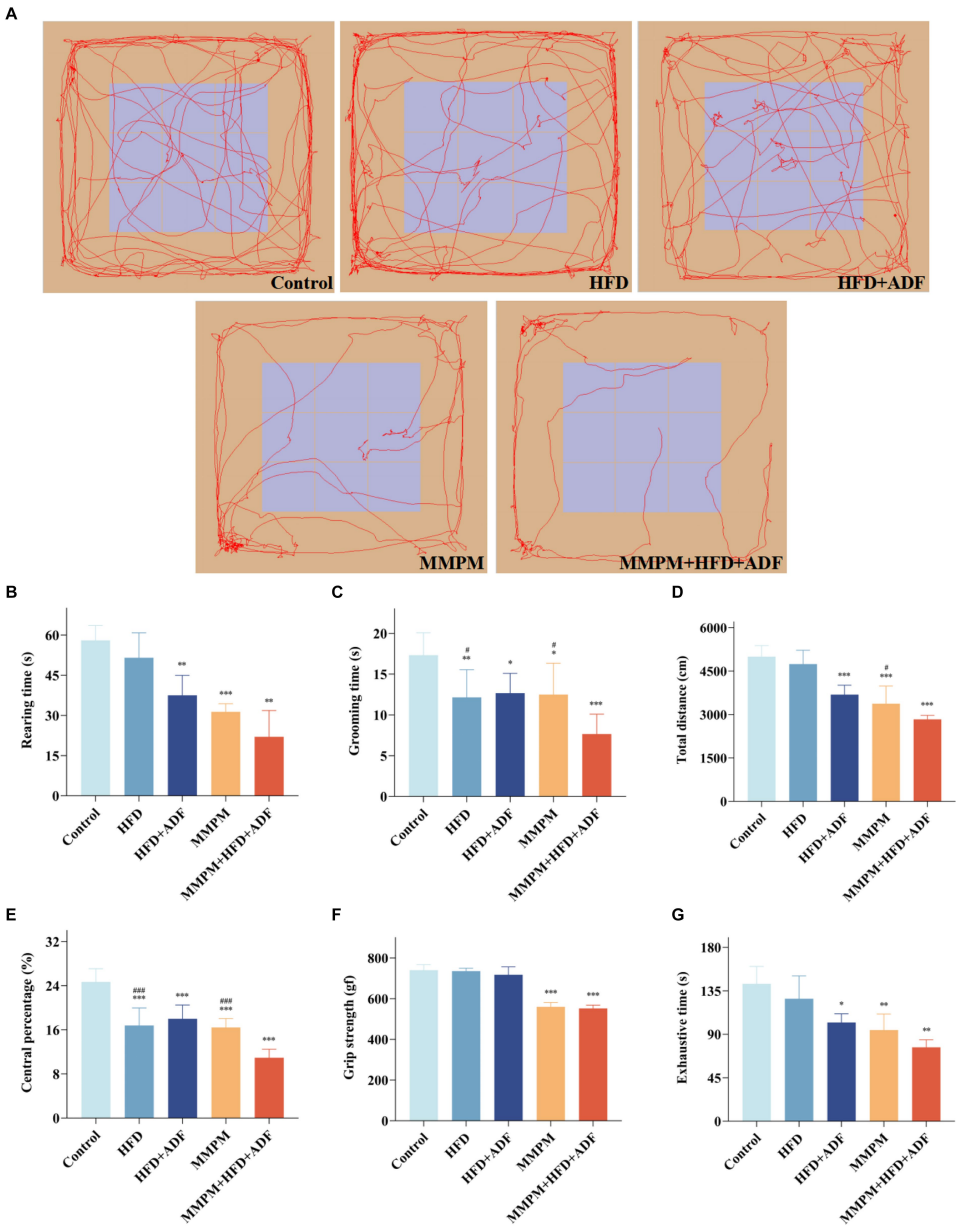
The locomotor activity decline and physical fatigue manifestations were evaluated through open field, grip strength, and exhaustive swimming tests **(A–E)** Results of the Open Field Experiment. **(A)** The map of representative movement trajectories in each group for 5 min in the open field, rearing time **(B)**, grooming time **(C)**, total distance **(D)**, central percentage **(E)**. **(F)** Grip strength test was performed to detect skeletal muscle fatigue of rats. **(G)** Forced exhaustive swimming test was conducted to test the exercise endurance and evaluate the level of fatigue. Data were expressed as (mean ± SD), *p < 0.05, **p < 0.01, ***p < 0.001 vs. control group (*n* = 6). ^#^*p* < 0.05, ^###^*p* < 0.001 vs. MMPM+HFD + ADF group (*n* = 6).

The results of the OFT revealed a significant decrease in vertical movements for the HFD + ADF group (*p* = 0.004), MMPM group (*p <* 0.001), and MMPM+HFD + ADF group (*p* = 0.001) compared to the control group (Figures 2B,C). For rearing duration, the HFD group (*p* = 0.006), HFD + ADF group (*p* = 0.013), MMPM group (*p* = 0.010), and MMPM+HFD + ADF group (*p <* 0.001) all exhibited significantly lower rearing times compared to the control group. Particularly noteworthy is the observation that the reduction in rearing time was more pronounced in the ADF + MMPM group compared to the HFD and conventional MMPM groups (*p* = 0.016, *p* = 0.010). Notably, when compared with the conventional MMPM group, the MMPM+HFD + ADF group exhibited a more obvious decrease in total distance traveled (*p* = 0.034; Figure 2D). The percentage of central zone movement distance for each group was notably lower than that of the control group (*p <* 0.001, *p <* 0.001, *p <* 0.001, *p <* 0.001; Figure 2E). Compared with the HFD group and the conventional MMPM group, the percentage of moving distance in the central region was significantly reduced in the MMPM+HFD + ADF group (*p <* 0.001, *p <* 0.001). The performance of rats in the MMPM+HFD + ADF group indicated a marked reduction in their exploratory drive and spontaneous activity within the open field arena The performance of rats in the MMPM+HFD + ADF group indicated a marked reduction in their exploratory drive and spontaneous activity within the open field arena (Figure 2A).

The grip strength test results revealed a significant reduction in grip strength for rats in both the MMPM and MMPM+HFD + ADF groups compared to the control group (*p <* 0.001, *p <* 0.001; Figure 2F). The results of FEST demonstrated a significant decrease in exhaustion time for the HFD + ADF group, MMPM group, and MMPM+HFD + ADF group compared to the control group (*p* = 0.015, *p* = 0.007, *p* = 0.001; Figure 2G). The aforementioned results indicate that the MMPM+HFD + ADF model may exhibit a decline in skeletal muscle fatigue and exercise endurance to rats.

### 3.2 The anomalous emotional states

**Figure 3:**
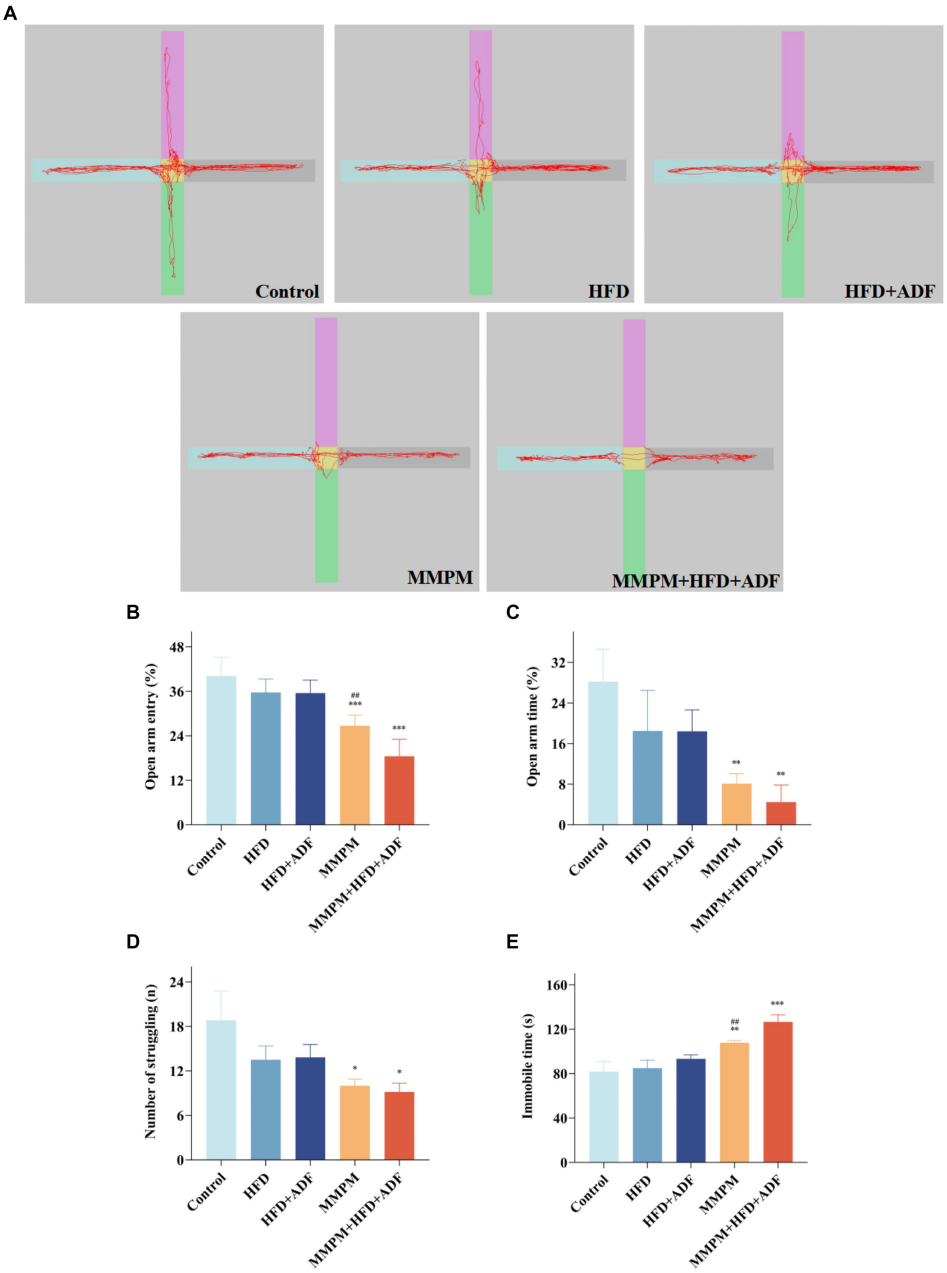
The assessment of emotional anomalies through elevated plus maze experiments and tail suspension tests. **(A–C)** Results of the Elevated Plus Maze Experiment. **(A)** The map of representative movement trajectories in each group for 5 min in the elevated plus maze. **(B,C)** Open arm entry and time of rats on EPM. **(D,E)** Results of the Tail Suspension Test. The number of struggling **(D)** and immobile time **(E)** of rats in tail suspension test are shown. Data were expressed as (mean ± SD), *p < 0.05, **p < 0.01, ***p < 0.001 vs. control group (*n* = 6). ^##^*p* < 0.01 vs. MMPM+HFD + ADF group (*n* = 6).

In this study, we utilized the elevated plus maze test to assess the anxiety level of the rats in each group. The findings revealed a significant decrease in the percentage of entries into the open arms (*p <* 0.001, *p <* 0.001; Figure 3B) and the percentage of time spent in the open arms (*p* = 0.003, *p* = 0.001; Figure 3C) for the MMPM group and the MMPM+HFD + ADF group of rats when compared with the control group, which indicated the presence of anxiety-like behavior and an increase in negative emotions in these group of rats (Figure 3A). It is still noteworthy that the percentage of entries into the open arms in the MMPM+HFD + ADF model was also significantly decreased when compared with the MMPM group (*p* = 0.001).

Furthermore, depressive-like behaviors were assessed in all rat groups using the tail suspension test. The results revealed that, compared to the control group, a significant decrease in struggling behavior was observed in the MMPM and MMPM+HFD + ADF groups (*p* = 0.022, *p* = 0.012; Figure 3D), and an immobility time that was significantly increased (*p* = 0.008, *p <* 0.001; Figure 3E). Notably, when compared with the traditional MMPM model, the rats in the MMPM+HFD + ADF model group also demonstrated a significant increase in the immobile time (*p* = 0.004), indicating that MMPM+HFD + ADF induced a notable manifestation of depressive-like behavior in rats.

### 3.3 Learning and memory function

**Figure 4:**
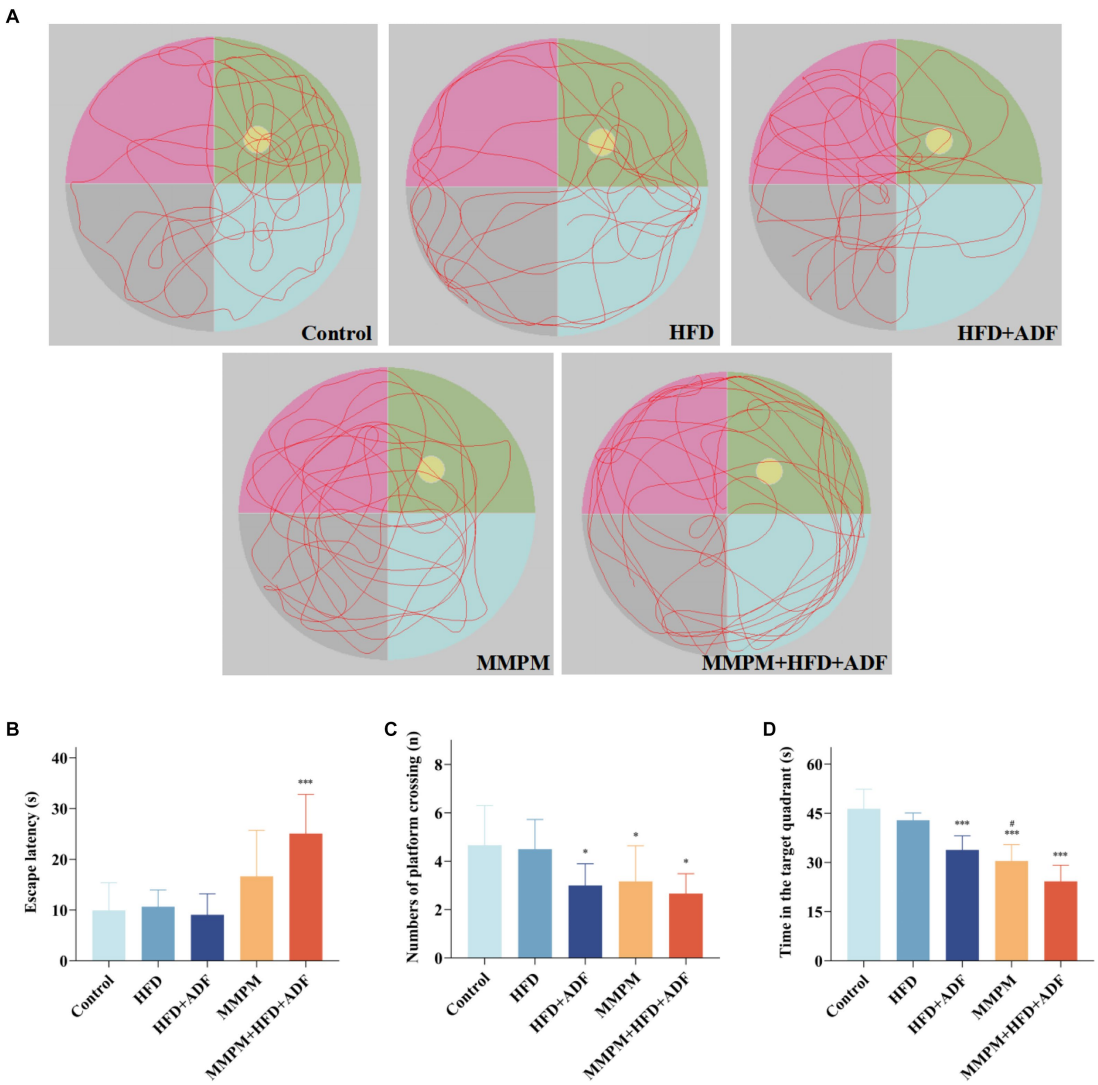
The evaluation of the cognitive function improvement was conducted through Morris water maze experiments. **(A)** Representative swim paths during the spatial probe test. Yellow circle represents the position of the platform, and green area represents the first quadrant, which is the target quadrant. The escape latencies **(B)**, numbers of platform crossings **(C)**, the time in target quadrant **(D)** on the last day during MWM test. Data were expressed as (mean ± SD), *p < 0.05, ***p < 0.001 vs. control group (*n* = 6). ^#^*p* < 0.05 vs. MMPM+HFD + ADF group (*n* = 6).

The MWM test was employed to assess the alterations in learning and memory function among the five group of rats. Figure 4A depicted the representative trajectories of rats during the memory exploration phase within a 2-min period. Compared with the control group, the rats in the HFD + ADF group, MMPM group, and MMPM+HFD + ADF group displayed significantly fewer crossing times through the platform area (*p* = 0.029, *p* = 0.048, *p* = 0.010; Figure 4C) and spent less time swimming in the target quadrant (*p <* 0.001, *p <* 0.001, *p <* 0.001; Figure 4D). When compared with the MMPM group, rats in the MMPM+HFD + ADF group showed a significantly reduced time spent swimming in the target quadrant (*p* = 0.029). Moreover, it is noteworthy that, in comparison to the control group, rats in the ADF + MMPM model exhibited a significant increase in escape latency (*p <* 0.001; Figure 4B) as well. These findings suggest a reduction in the learning and memory capabilities of rats in the HFD + ADF, MMPM, and MMPM+HFD + ADF groups. Among them, the MMPM+HFD + ADF group demonstrated the most significant decline in learning and memory abilities.

### 3.4 Biochemical indicators

**Figure 5:**
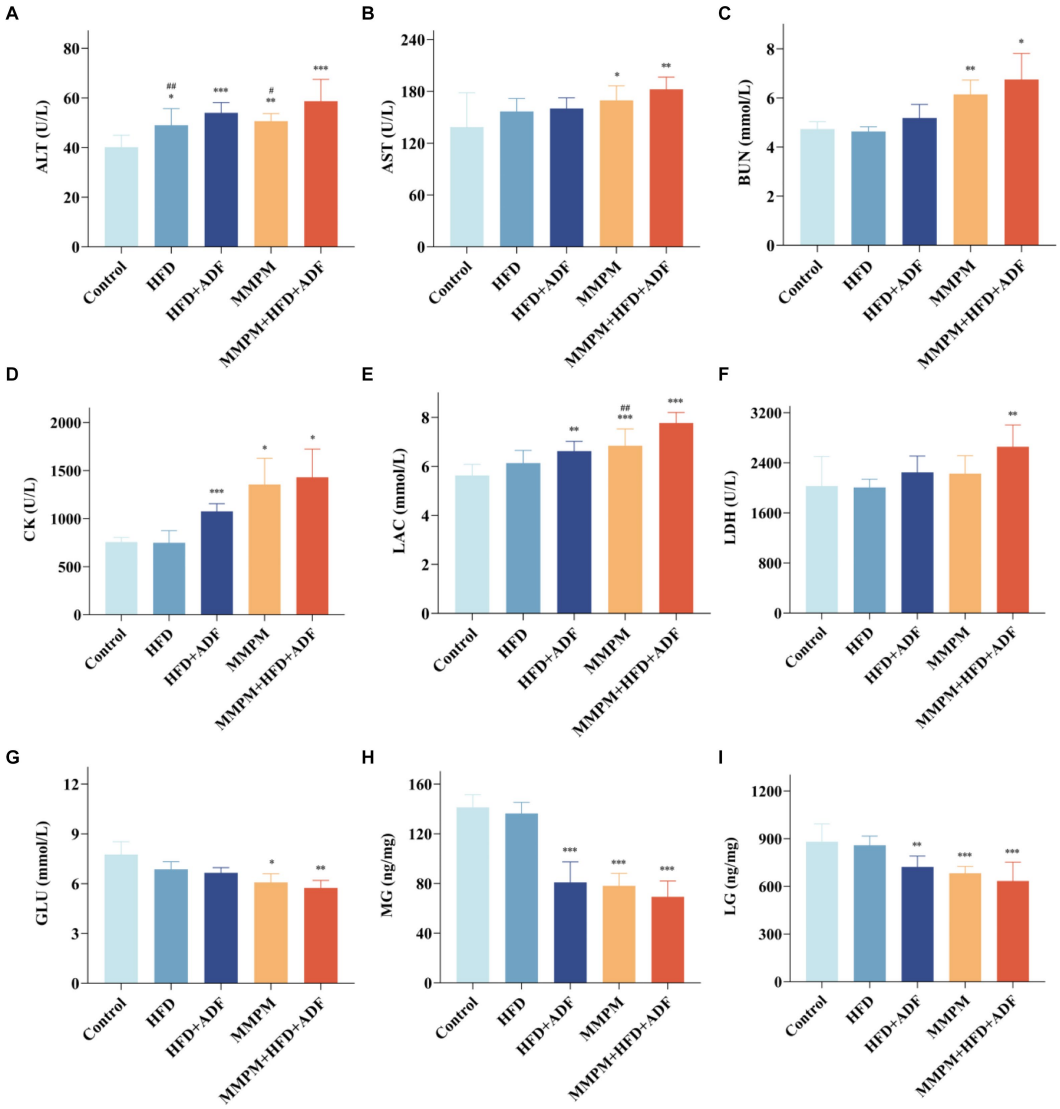
The results of biochemical parameter analysis related to fatigue. **(A–G)** Serum levels of ALT, AST, BUN, CK, LAC, LDH and GLU were measured with an automatic biochemical analyzer. **(H,I)** The expression levels of MG and LG were tested by ELISA kits. Data were expressed as (mean ± SD), *p < 0.05, **p < 0.01, ***p < 0.001 vs. control group (*n* = 6). ^##^*p* < 0.01 vs. MMPM+HFD + ADF group (*n* = 6).

In this study, we tested the levels of serum ALT, AST, BUN, CK, LAC, LDH, and GLU, which are commonly employed as the indicators for evaluating fatigue, in the peripheral blood of different groups of rats (Boyas and Guével, 2011; Tian et al., 2022). The results revealed a significant increase in serum ALT levels in the peripheral blood of rats in the four model groups compared to the control group (*p* = 0.015, *p <* 0.001, *p* = 0.005, *p <* 0.001; Figure 5A). Specifically, in the MMPM+HFD + ADF model group, the ALT levels were significantly higher than those in the HFD group (*p* = 0.008) and the MMPM group (*p* = 0.026). Compared to the control group, the MMPM and MMPM+HFD + ADF groups exhibited significantly elevated levels of AST (*p* = 0.022, *p* = 0.002; Figure 5B) and BUN (*p* = 0.008, *p* = 0.045; Figure 5C), while showing a significant decrease in GLU levels (*p* = 0.018, *p* = 0.006; Figure 5G). Furthermore, in comparison to the control group, the HFD + ADF, MMPM, and MMPM+HFD + ADF groups demonstrated increased levels of CK (*p <* 0.001, *p* = 0.026, *p* = 0.023; Figure 5D) and LAC (*p* = 0.002, *p <* 0.001, *p <* 0.001; Figure 5E). Particularly noteworthy was the more pronounced elevation of LAC levels in the MMPM+HFD + ADF group compared to the MMPM group (*p* = 0.004). Additionally, in comparison to the control group, only the MMPM+HFD + ADF group exhibited a significant elevation in LDH levels (*p* = 0.002; Figure 5F).

The levels of glycogen in skeletal muscle and liver serve as reflective indicators of overall bodily energy expenditure. Our results revealed that the levels of LG (*p* = 0.004, *p <* 0.001, *p <* 0.001; Figure 5I) and MG (*p <* 0.001, *p <* 0.001, *p <* 0.001; Figure 5H) were significantly reduced in the HFD + ADF group, MMPM group, and MMPM+HFD + ADF group as compared to the control group.

### 3.5 Pathological morphological changes in the hippocampal CA1 region

**Figure 6:**
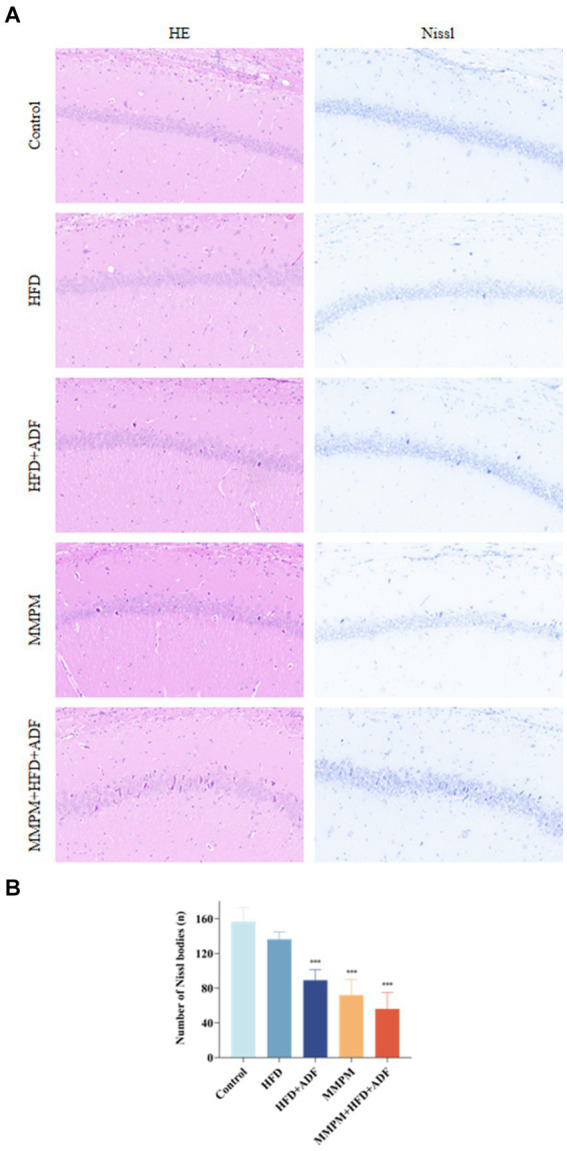
Pathological changes of rat hippocampus CA1 region are observed. **(A)** HE staining and Nissl staining are shown (magnification ×200). **(B)** The number of Nissl bodies. The Nissl bodies were stained blue. Three fields were selected randomly in each sample for Nissl bodies calculation, and the numbers of Nissl bodies were averaged. Data were expressed as (mean ± SD), ***p < 0.001 vs. control group (*n* = 3).

Figure illustrates the morphological changes in the hippocampal tissue of rats from different groups. In the control group and the high-fat diet (HFD) group, the hippocampal tissue structure was fundamentally normal, characterized by a tightly and orderly arranged neuronal cell pattern, with only a very few instances of neuronal degeneration observed. In the hippocampal tissue of rats in the HFD + ADF and MMPM groups, a mild abnormality is evident, characterized by a slight degeneration of neuronal cells. However, the pathological alterations in the hippocampal tissue of rats in the MMPM+HFD + ADF model are most pronounced, with a substantial degeneration of neuronal cells, prominent nuclear condensation, and intensified cellular basophilia (Figure 6A). Nissl staining revealed a significant decrease in the number of Nissl bodies in the hippocampal tissue of rats in the HFD + ADF, MMPM, and MMPM+HFD + ADF groups (*p <* 0.001, *p <* 0.001, *p <* 0.001; Figure 6B), indicating impaired neuronal function.

### 3.6 Alterations in the ultrastructure of mitochondria in the hippocampal CA1 region

**Figure 7:**
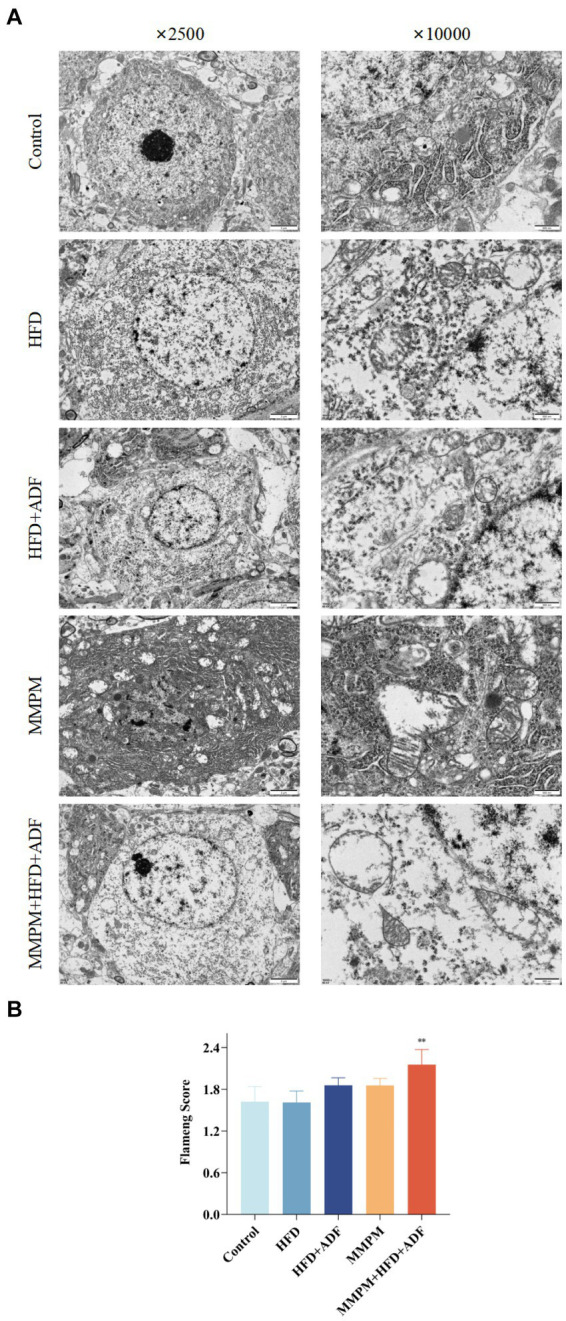
Observation of changes in the hippocampal CA1 region using transmission electron microscopy. **(A)** The ultrastructure of mitochondria in hippocampus CAI region, are observed by electron microscope (magnification ×2,500, magnification ×10,000). **(B)** Flameng score. Three fields were selected randomly in each sample, and 10 mitochondria were observed in each field. The mitochondrial Flameng score of each sample was calculated as the mean of the 30 mitochondrial total scores. Data were expressed as (mean ± SD), **p < 0.01 vs. control group (*n* = 3).

The occurrence of central fatigue is closely associated with cerebral energy metabolism, and the morphological structure of mitochondria can directly reflect whether mitochondrial function is normal. We employed transmission electron microscopy method to further investigate the occurrence of mitochondrial ultrastructural damage in the CA1 region of the hippocampus in various groups of rats (Figure 7A). In the control group of rats, the mitochondria in the hippocampus displayed regular morphology with intact double membrane structures and neatly arranged cristae. In the HFD group, a mild swelling of mitochondria with blurred structure was observed. Both the HFD + ADF group and the MMPM group showed a reduction in mitochondrial quantity in the cytoplasm, with a significant swelling of mitochondria and unclear double membrane structures. In the MMPM+HFD + ADF group, some nerve fibers exhibited thinning of myelin sheaths and even fragmentation, accompanied by unclear internal mitochondrial cristae, disordered arrangement, disrupted double membrane structures, and more pronounced irregular morphological changes within the mitochondria. Furthermore, based on the Flameng score, we could only observe a significant difference in the MMPM+HFD + ADF group compared to the control group (*p* = 0.003; Figure 7B), indicating the most severe mitochondrial damage in this group.

### 3.7 The hippocampal tissue of rats exhibited abnormal neurotransmitter secretion, disrupted energy metabolism, and oxidative stress damage

**Figure 8:**
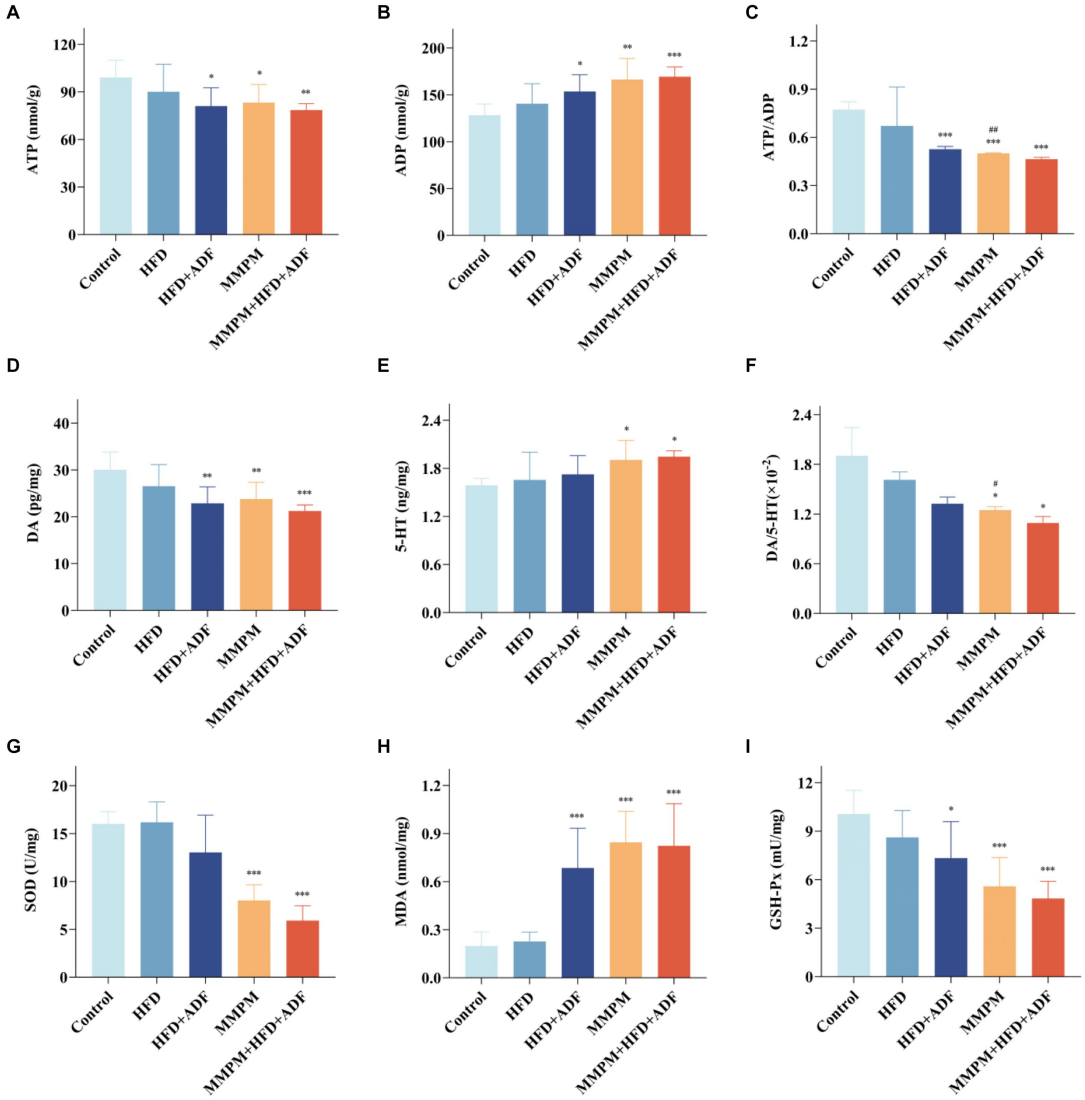
Observed manifestations of neurotransmitter secretion abnormalities, energy metabolism disruption, and oxidative stress damage in hippocampal. **(A–F)** The expression levels of ATP, ADP, DA, 5-HT in rat hippocampus were tested by ELISA kits and the ratio of ATP to ADP and DA to 5-HT are shown. **(G–I)** The expression levels of SOD, MDA, GSH-Px. Data were expressed as (mean ± SD), *p < 0.05, **p < 0.01, ***p < 0.001 vs. control group (*n* = 6). ^##^*p* < 0.01 vs. MMPM+HFD + ADF group (*n* = 6).

5-HT and DA are a pair of neurotransmitters in the brain that are currently believed to play a significant role in the development of central fatigue (Yang et al., 2019). An increase in the ratio of 5-HT to DA can accelerate the onset of central fatigue (Meeusen and Watson, 2007). Our results indicated a significant increase in 5-HT levels in the hippocampal tissue of rats in the MMPM and MMPM+HFD + ADF groups (*p* = 0.021, *p* = 0.010; Figure 8E). While, the levels of DA in the hippocampal tissue of rats in the HFD + ADF group, MMPM group, and MMPM+HFD + ADF group showed a significant decrease (*p* = 0.002, *p* = 0.006, *p <* 0.001; Figure 8D). The ratio of DA/5-HT was significantly decreased in the MMPM group, and MMPM+HFD + ADF group (*p* = 0.048, *p* = 0.016; Figure 8F).

ATP serves as a crucial product of energy metabolism (Li et al., 2016). The alteration in the ratio of ATP/ADP serves as one of the indicators of changes in energy metabolism (Amantini et al., 2016). Our findings showed that compared with the control group, the ATP levels decreased in the HFD + ADF group, MMPM group, and MMPM+HFD + ADF group (*p* = 0.014, *p* = 0.028, *p* = 0.006; Figure 8A), while the ADP levels increased (*p* = 0.019, *p* = 0.001, *p <* 0.001; Figure 8B). The ratio of ATP/ADP was significantly decreased in the HFD + ADF group, MMPM group, and MMPM+HFD + ADF group (*p <* 0.001, *p <* 0.001, *p <* 0.001; Figure 8C).

**Figure 9:**
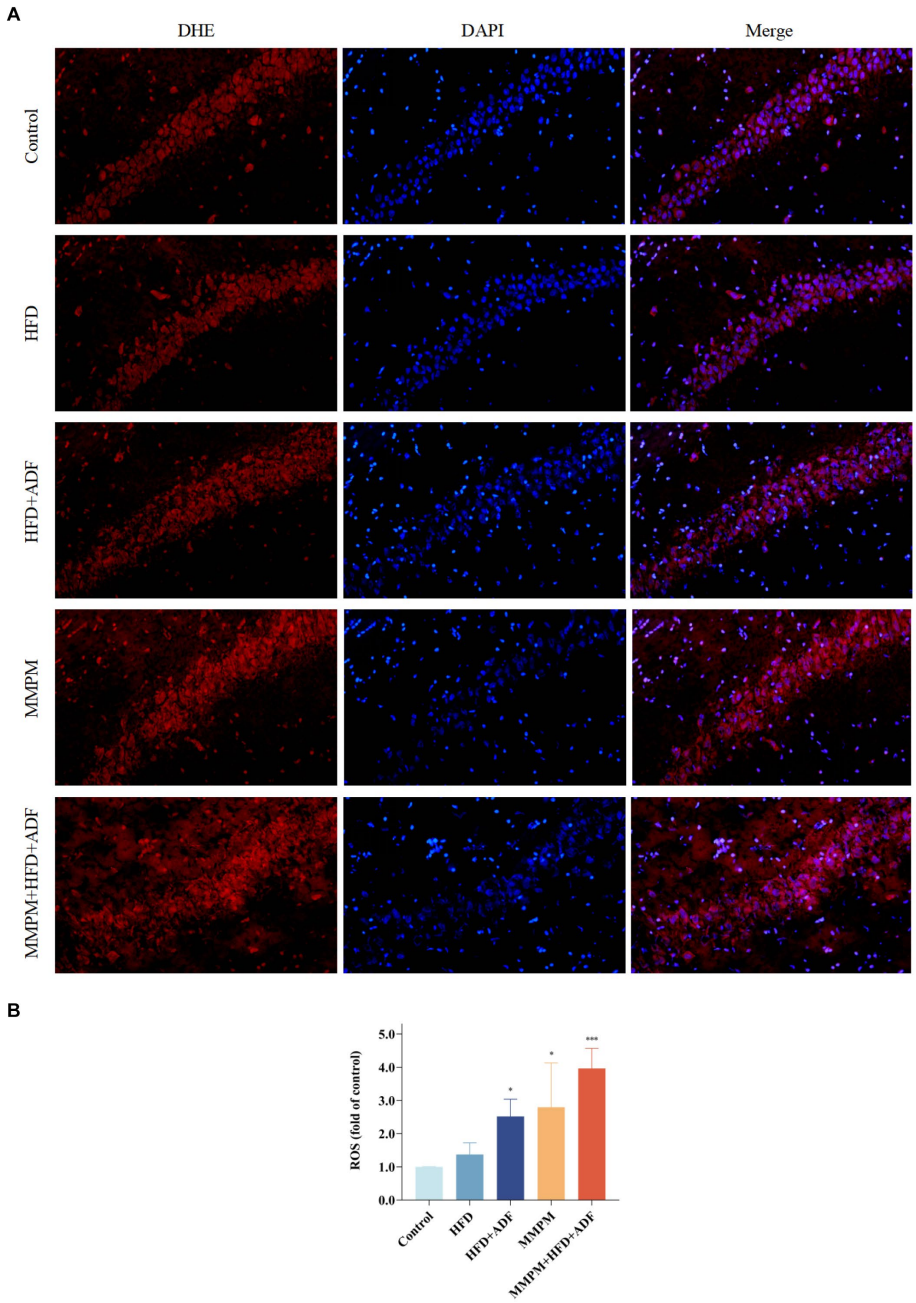
Level of reactive oxygen species (ROS) in CA1 region of rat hippocampus. **(A)** The Microscopy images (magnification ×200). **(B)** The statistical results of intracellular ROS generation in CA1 region of rat hippocampus. The nuclei in the frozen section were interpreted as blue by DAPI staining, while the intracellular ROS was stained red with DHE. The average light density value of the red fluorescence can represent the relative ROS content. The value of relative ROS content was normalized to the control group and expressed as fold of the control group. Data were expressed as (mean ± SD), *p < 0.05, ***p < 0.001 vs. control group (*n* = 3).

Moreover, we also assessed the levels of SOD, MDA, GSH-Px, and ROS to reflect the oxidative stress damage in the hippocampal tissues of rats in each group. Our results revealed that the SOD activity in the hippocampal tissues of rats in the MMPM group and MMPM+HFD + ADF group showed a significant decrease (*p <* 0.001, *p <* 0.001; Figure 8G) when compared to the control group. Furthermore, the GSH-Px activity in the HFD + ADF group, MMPM group, and MMPM+HFD + ADF group exhibited a significant reduction (*p* = 0.010, *p <* 0.001, *p <* 0.001; Figure 8I) when compared to the control group. Meanwhile, compared to the control group, the MDA and ROS levels in the HFD + ADF group, MMPM group, and MMPM+HFD + ADF group significantly increased (*p <* 0.001, *p <* 0.001, *p <* 0.001; Figure 8H; *p* = 0.027, *p* = 0.012, *p <* 0.001; Figures 9A,B).

## 4 Discussion

Central fatigue is increasingly recognized as a significant health condition in modern society, significantly impacting the quality of life and work efficiency (Luo et al., 2022). In our previous studies, we have found that by incorporating disruptions in sleep patterns and dietary rhythms into the modeling of central fatigue, specifically utilizing the MMPM in conjunction with ADF methods, we have been able to preliminarily establish an animal model of central fatigue that closely mirrors human characteristics (Zhang et al., 2024). Prior studies have confirmed that HFD can impact the metabolic balance of the body and the normal function of the central nervous system. In this study, we chose a high-fat feeding method for rats that exhibited more pronounced effects on dietary patterns and metabolism. We found that the method of ADF combined with HFD partial mimicked central fatigue and metabolic disturbances in rats. The integration of this HFD + ADF method with the MMPM model exemplifies a promising and versatile animal model for central fatigue. In contrast to the conventional MMPM model of central fatigue, this model demonstrates heightened pathological traits with regards to adverse emotional states, cognitive function, and oxidative stress damage in rats.

### 4.1 The HFD + ADF method can induce partial central fatigue symptoms in rats

#### 4.1.1 Feeding with HFD alone for 21 days did not lead to the anticipated changes of rats

Currently, evidence from clinical and investigative studies has shown a close association between dietary factors and fatigue (Oberle et al., 2020; Haß et al., 2019). Unfavorable dietary habits, such as prolonged consumption of a high-fat diet and alterations in eating patterns, have been found to be correlated with the occurrence of fatigue (Nosaka et al., 2020). Nevertheless, current research has yet to demonstrate a relationship between dietary factors and the onset of central fatigue, nor have studies investigated the manifestations of central fatigue-related symptoms in rats in dietary interference animal models, or assessed biochemical markers associated with fatigue in their serum. In this study, we investigated the common central fatigue-related behavioral manifestations, serum biochemical markers, and brain-related damages in rats subjected to high-fat feeding and alternate-day feeding combined with high-fat feeding. We observed that the simple HFD model did not induce significant changes in the central fatigue-related behavioral manifestations, biochemical markers, and brain-related damages in rats. Only in the ALT parameter was a significant increase observed in the HFD group, which may be linked to a marked increase in hepatic metabolic burden due to the high-fat diet (Liou et al., 2019). The likely reason for these results may be attributed to the relatively short modeling period in this study. HFD has been shown to impact the emotional and cognitive functions of rodent animals and induce significant changes in the hippocampal tissue, a process that may require a modeling period of 8 weeks or even longer (Mota et al., 2023).

#### 4.1.2 ADF can expedite the central fatigue related changes of HFD on the rats

In contrast to feeding with HFD alone, rats in the HFD + ADF group exhibited abnormal behaviors, such as significant reductions in exercise endurance, decreased locomotor activity, diminished exploratory behavior, and impaired learning and memory abilities. These alterations may be attributed to the disruptive effects of irregular dietary patterns on the peripheral motor system and brain memory functions. The confluence of ADF and HFD elements appears to have expedited the onset of central nervous system impairment and fatigue in the rats. It was noted that the HFD + ADF group exhibited a notably heightened food intake on designated feeding days compared to the control group, effectively emulating an erratic dietary schedule. These findings are consistent with clinical study results indicating that irregular dietary patterns have the potential to affect energy metabolism and the overall health status of the body (Farshchi et al., 2004). However, discernible emotional perturbations were not detected in the HFD + ADF group during the tail suspension and elevated plus maze tests. Prior investigations have indicated that HFD exposure can trigger deleterious emotional repercussions in rats, such as heightened anxiety and depressive tendencies, albeit typically manifesting over extended durations spanning 14 to 40 weeks (Kurhe and Mahesh, 2017; Griffin et al., 2010). This indicates that the HFD + ADF modeling method is still insufficient to simulate the emotional manifestations of central fatigue within a 3-week modeling period.

In the HFD + ADF group, significant alterations were also observed in five classic fatigue-related metabolic and biochemical markers: ALT, CK, muscle glycogen, liver glycogen, and lactate. Rats in the HFD + ADF group exhibited markedly elevated levels of ALT, CK, and lactate, while levels of muscle glycogen and liver glycogen were significantly reduced. Numerous studies have demonstrated that poor dietary habits can induce significant metabolic alterations in the liver and muscles, leading to elevated levels of ALT, CK, and lactate, as well as changes in the storage and availability of energy metabolic substrates (glycogen) (Albouery et al., 2021; Dillon et al., 2009; Hackney and Viru, 2008; Tomizawa et al., 2014; Penney et al., 2020). Mild alterations in tissue morphology and mitochondrial structure were observed in the hippocampal CA1 region of rats in the HFD + ADF group, accompanied by reduced mitochondrial function and occurrence of oxidative stress damage. DA is one of the crucial neurotransmitters in central nervous system, with fatigue often being associated with a decline in dopaminergic system function (Brown et al., 2016; Zhao et al., 2022). We observed a significant decrease in hippocampal DA levels in the rats of HFD + ADF group. These findings indicated that HFD + ADF can lead to the manifestation of central fatigue symptoms and alterations in markers in rats. In contrast to the simple ADF modeling method, the HFD + ADF approach not only fails to enhance the health of rats but also induces metabolic disorders and neuronal damage (Xu et al., 2010; Bhoumik et al., 2022). It is believed that the combination of HFD and ADF factors is likely to result in a state of severe dietary irregularity for the rats, which does not foster health but rather augments the risk of bodily homeostasis imbalance. Furthermore, it may contribute to the emergence of partial central fatigue symptoms (Bilibio et al., 2023).

### 4.2 The MMPM method can induce oxidative stress damage in the hippocampus of central fatigue rats

The MMPM method has been established as a classic and effective means of creating an animal model of central fatigue through sleep disturbance (Han C, et al., 2017; Dong et al., 2017). The reliability of the central fatigue model induced by the MMPM method has been evaluated and investigated to date regarding behavioral testing, serum indicators of fatigue, and alterations in brain neurotransmitters (Han C, et al., 2017; Li et al., 2024; Xu et al., 2022). However, research examining oxidative stress levels in rats with central fatigue models constructed using the MMPM method is lacking. Oxidative stress can lead to mitochondrial damage and dysfunction in the hippocampal tissue (Li et al., 2022). Both this study and our previous research have confirmed the presence of mitochondrial structural damage in the hippocampus of rats in the central fatigue model of MMPM, which may be attributed to oxidative stress. Moreover, researchers have indicated that the oxidative stress damage can lead to the occurrence of mental fatigue (Imai et al., 2018; Huang et al., 2011). Mental fatigue is a crucial cognitive element of central fatigue, characterized by a state of cortical inactivation resulting in decreased cognitive performance and diminished alertness (Chunlin et al., 2017). Our research findings indicated a significant increase in the levels of oxidative indicators ROS and MDA, and a notable decrease in the activities of antioxidant indicators SOD and GSH-Px in the hippocampal tissue of rats in the MMPM model, suggesting the presence of significant oxidative stress damage in the hippocampal tissue. Therefore, may serve as one of the characteristic pathological damages in the brains of MMPM central fatigue model rats, and further research is needed to explore the specific underlying mechanisms.

### 4.3 The method of MMPM+HFD + ADF can induce the most prominent central fatigue manifestations

Given that both dietary and sleep factors are significant contributors to fatigue, traditional MMPM models predominantly consider the impact of sleep factors (Zhang et al., 2018). Focusing solely on dietary regularity fails to replicate the emotional manifestations of central fatigue. Our study, building upon the MMPM model, introduced an HFD + ADF regimen to alter the type of food intake and normal dietary rhythm of rats, thereby intensifying the impact of dietary factors. This approach aimed to better simulate irregular eating patterns in real life and construct an animal disease model that more accurately mirrors the clinical features of central fatigue onset.

#### 4.3.1 Rats in the MMPM+HFD + ADF model display remarkable central fatigue-related behavioral changes

Central fatigue often manifests as emotional distress, cognitive impairment, and physical fatigue-related symptoms in clinical (Lee et al., 2020; Han C, et al., 2017). In rodent models, central fatigue can manifest significant changes in physical fatigue, negative emotions, and autonomous activity during behavioral tests (Zhang et al., 2024). Our findings indicate that, regarding behavioral outcomes, the MMPM+HFD + ADF model rats demonstrated more pronounced declines in autonomous movement capabilities and diminished exploratory drive compared with those in the conventional MMPM model group. Additionally, they displayed heightened anxiety and depressive symptoms, as well as significant impairments in learning and memory functions.

#### 4.3.2 The MMPM+HFD + ADF model rats exhibit significant alterations in fatigue-related serum biochemical metabolic indicators

The findings from the analysis of serum biochemical metabolic indicators demonstrated notable changes in fatigue-related parameters in the MMPM+HFD + ADF model rats, suggesting substantial energy expenditure, reduced glycogen reserves, and disrupted glucose metabolism in these rats. Among the concerns is the levels of LAC in the peripheral blood of rats in the MMPM+HFD + ADF model were significantly elevated compared to those in the MMPM model. A significant increase in LDH levels in the peripheral blood was observed only in rats in the MMPM+HFD + ADF model when compared to the control group. These results indicated the most severe lactate accumulation in rats of the MMPM+HFD + ADF model compared with the other models. Blood lactate level is one of the key indicators for assessing fatigue (Gupta et al., 2015). Accumulation of intracellular lactate can lead to the onset of fatigue (Zhang et al., 2020). LDH plays a role in controlling lactate formation and regulating lactate turnover (Venojärvi et al., 2005). It can also serve as a crucial indicator for assessing the level of fatigue (Yang et al., 2022). Thus, the aforementioned findings suggested that the MMPM+HFD + ADF model in rats exhibited the most comprehensive and significant fatigue-related characteristics.

#### 4.3.3 The MMPM+HFD + ADF model rats exhibit notable changes in hippocampal

An increasing body of evidence suggests that the brain serves as a central regulator of fatigue (Seguro et al., 2022). Regarding the pathological morphology of the hippocampal CA1 region, the pathological changes in the hippocampal tissue of rats in the MMPM+HFD + ADF model were particularly pronounced. A significant reduction in Nissl bodies was observed, and mitochondrial ultrastructure damage was notably severe, indicating substantial neuronal and mitochondrial damage within the hippocampus of rats in the MMPM+HFD + ADF model. Mitochondria are the primary cellular organelles responsible for ATP production, and a decrease in ATP levels across various organs and tissues directly contributes to fatigue (Xu et al., 2017). Furthermore, we observed a severe imbalance in brain ATP/ADP, DA/5-HT levels, alongside notable oxidative stress damage in rats from the MMPM+HFD + ADF group. Therefore, the above findings confirmed that the central fatigue rat model established by the MMPM+HFD + ADF method can exhibit a greater and more pronounced array of central fatigue pathophysiological features.

While our study sheds light on important findings, it is crucial to acknowledge the limitations that warrant consideration. Subsequent investigations should delve deeper into elucidating the efficacy of the innovative model established through the integration of MMPM, HFD, and ADF methodologies in faithfully reproducing critical facets of genuine clinical scenarios, including the rhythmicity of dietary consumption timing and its repercussions on the peripheral motor system.

## 5 Conclusion

In summary, our findings suggested that the dietary intervention modeling approach utilizing HFD + ADF method leads to the manifestation of symptoms indicative of central fatigue in rats. Meanwhile, the multifactorial composite model involving MMPM+HFD + ADF method yields a more robust and clinically relevant model of central fatigue in rats. Additionally, significant oxidative stress alterations in the brain of rats in the central fatigue model indicate it as a potential key factor contributing to the manifestation of central fatigue-related symptoms. The findings of our study provide a more reliable animal model and theoretical foundation for central fatigue research. This animal model holds profound application value for the study of the pathophysiological mechanisms of central fatigue and the pharmacological research of therapeutic drugs.

## Data Availability

The raw data supporting the conclusions of this article will be made available by the authors, without undue reservation.
